# SBOA-Inspired Particle Swarm Optimization of XGBoost for False Data Injection Detection in P2P Energy Trading

**DOI:** 10.3390/s26196342

**Published:** 2026-10-08

**Authors:** Wenxuan Xu, Koichi Kobayashi, Yuh Yamashita

**Affiliations:** Graduate School of Information Science and Technology, Hokkaido University, Sapporo 060-0814, Japan

**Keywords:** P2P energy trading, false data injection, XGBoost, particle swarm optimization, secretary bird optimization, hyperparameter optimization

## Abstract

Peer-to-peer (P2P) energy trading depends on the integrity of smart-meter measurements and coordinator messages. This paper presents an XGBoost detector for false data injection attacks and a hybrid hyperparameter-search mechanism combining particle swarm optimization (PSO) with three hunting-inspired operators from the secretary bird optimization algorithm (SBOA). A common candidate-evaluation budget enables comparison of fixed XGBoost, PSO-XGBoost and SBOA-PSO-XGBoost. The evaluation covers four Austin dataset settings, two attack scenarios and nine classifiers, using accuracy, precision, recall, F1 and computational cost. The equal-scenario mean F1 scores are 90.06%, 94.47% and 96.94% for the three XGBoost variants, respectively. The hybrid adds 2.47 percentage points over PSO with a 5.16% increase in total computation time, while its batch-prediction cost remains close to that of PSO-XGBoost. These comparisons characterize the detection–cost trade-off and the role of hybrid search under a shared evaluation protocol.

## 1. Introduction

A smart grid is a next-generation power supply system that optimizes the amount of electricity used by forming a bidirectional power network that can be controlled from both the supply side and the consumption side, unlike conventional power supply systems [1,2,3]. In a conventional power system, electricity is generated at a small number of large centralized plants and is delivered unidirectionally to consumers. Since the supply side has little information on how much electricity is actually consumed and when, a large capacity margin must be reserved in advance, which results in inefficient operation. In contrast, a smart grid incorporates information and communication technologies into every layer of the power network, and thus measurement data, control commands, and price signals are exchanged bidirectionally among generators, aggregators, and consumers. Such an architecture also facilitates the large-scale introduction of distributed energy resources (DERs), such as photovoltaic panels, wind turbines, storage batteries, and electric vehicles, whose outputs are intermittent and difficult to predict. Through the digitalization of the power grid, new technologies such as smart meters and demand response are utilized. Smart meters are highly capable electricity meters and combine power measurement and communication functions [4,5,6]. Unlike conventional analog meters, which require periodic manual reading and provide only the cumulative amount of electricity consumed, smart meters record the consumption at a fine time granularity and transmit it automatically to the supply side through a communication network. In other words, smart meters are a kind of smart/intelligent sensors [7,8,9,10,11]. With smart meters, it is possible for the consumption side to send the supply side information such as the amount of electricity used per day and the relationship between time of day and power consumption. Such fine-grained data allows the supply side to estimate the demand more accurately, to detect faults and outages more quickly, and to design tariffs that reflect actual usage patterns. It is expected to reduce energy loss; for example, by decreasing the supply volume during periods of low power consumption. In addition, consumers can monitor their own consumption in almost real time, which motivates them to revise their electricity usage behavior. Demand response is a technology where electricity is controlled by changing the electricity usage of consumers by manipulating the electricity price, the incentive, and so on [12,13,14]. Demand response programs are broadly classified into price-based programs, such as time-of-use pricing and real-time pricing, and incentive-based programs, in which consumers receive a reward for curtailing their loads upon request. By shifting or curtailing flexible loads, such as air conditioners, water heaters, and electric vehicle charging, from peak periods to off-peak periods, demand response flattens the demand curve and reduces the need for costly peaking generators. This contributes to the stable operation of a power system with a high penetration of renewable energy sources. Among various technologies in a smart grid, we focus on peer-to-peer (P2P) energy trading. In P2P energy trading, prosumers who own DERs directly trade their surplus electricity with neighboring consumers through a local market platform, instead of selling it to a centralized utility.

P2P energy trading has attracted significant attention as a decentralized energy management model in the modern smart grid paradigm. In this model, network participants, known as prosumers, can directly share surplus energy resources with one another through a local market mechanism, which may include a coordinating agent. One of the primary drivers for this shift is economic efficiency: prosumers that need energy can purchase it from peers at prices lower than the main grid’s retail rate, while those with excess generation, typically from rooftop solar panels, can achieve higher financial returns compared to conventional schemes [15,16,17,18]. However, the rapid integration of distributed energy resources (DERs) introduces significant cyber-physical vulnerabilities. Many P2P trading platforms rely on home energy management systems (HEMS) and smart meters connected via the public Internet, which may often lack robust protection.

This exposure makes the system a prime target for malicious actors. Correct data preservation is important for ensuring the security and fairness of energy trading [19,20,21,22]. Any corruption of the data exchange process can lead to severe consequences, including grid imbalances, financial losses for participants, and a general loss of trust in decentralized management. Detecting malicious tampering with smart meter measurement data is of great importance for enhancing the reliability of sensors [23,24].

The loss of data integrity in energy markets is frequently caused by sophisticated cyber-attacks, such as False Data Injection Attacks (FDIAs), replay attacks, and man-in-the-middle attacks. Among these, FDIAs are particularly dangerous because they involve the subtle manipulation of measurement data or bidding information to deceive the market operator. Existing research has extensively studied the impact of FDIAs on traditional power systems. For instance, studies have shown how FDIAs can result in the rescheduling of generation, unnecessary load shedding, or even widespread blackouts through resonance attacks on load frequency control. In electricity markets, attackers have been shown to manipulate locational marginal prices to gain illegal financial advantages. Despite these advancements, a critical research gap remains: few studies investigate the impact of FDIAs specifically within a local P2P energy trading market involving prosumers. The existence of prosumers adds a layer of complexity because their dual role as both buyers and sellers challenges traditional utility-based defense mechanisms. Previous research has indicated that static attacks—those occurring before a trading game starts—might reduce prosumer profits, but they often fail to provide the attacker with “free energy.” This is because the iterative nature of trading games causes them to eventually converge to a supply-demand balance. This necessitates the study of dynamic, iterative attack models that can persist throughout the trading process.

To defend against these threats, researchers have explored both physical and cyber-oriented approaches. Physical security, such as locking measurement devices, is often cost-intensive and difficult to implement at scale across thousands of private households. Consequently, data-driven machine learning (ML) methods have gained traction as an efficient, cyber-oriented defense strategy. Conventional ML algorithms, including Naive Bayes (NB) [25], Decision Tree (DT) [26,27], K-Nearest Neighbors (KNN) [28], Support Vector Machine (SVM) [29,30], Random Forest (RF) [31,32], Multi-Layer Perceptron Neural Network (MLPNN) [33,34], and eXtreme Gradient Boosting (XGBoost) [35,36], have been applied to detect anomalies in power grid data. However, the application of high-performance ML models as a specific defense strategy in P2P energy trading is still in its infancy. Moreover, standard black-box models often suffer from a lack of interpretability or require extensive hyperparameter tuning to be effective in complex, dynamic trading environments.

The study distinguishes classifier choice from hyperparameter-search design. Fixed-parameter learners provide a reference for detection performance, while PSO-XGBoost isolates the contribution of standard swarm-based tuning. Comparing PSO-XGBoost with its hybrid variant under the same candidate-evaluation budget then focuses on the additional proposal operators. This structure separates the effect of tuning XGBoost from the incremental effect of modifying the search mechanism.

The two FDIA settings follow Mohammadi et al. [37,38]. The proposed detector combines XGBoost with SBOA-inspired PSO. The original SBOA models hunting and predator evasion [39]; this hybrid adapts its three hunting operators within the PSO update and omits the separate predator-escape stage. The swarm retains personal-best and global-best memories while alternating standard PSO proposals with the hunting-inspired candidates.

The main contributions are as follows:

1. A specified hybrid PSO search with an eight-dimensional XGBoost parameter encoding, a training-only validation objective, bounded proposals, and an explicit accounting of candidate evaluations.
2. A four-setting comparison of seven fixed-parameter classifiers, PSO-XGBoost and SBOA-PSO-XGBoost using accuracy, attack-class precision, recall, F1, error counts and computational cost.
3. An analysis of the quality–cost trade-off, class-dependent detection errors and their implications for deploying the detector.

A preceding conference paper [40] introduced the general PSO–XGBoost direction. The present work extends that framework through the SBOA-inspired proposal mechanism, a common search budget, scenario-specific feature definitions and an expanded comparison of detection performance and computational cost.

Section 2 describes the P2P system, and Section 3 describes the threat settings. Section 4 defines the detector and optimizer. Section 5 and Section 6 specify the evaluation protocol and results. Section 7 discusses the research contributions and their implications for P2P energy trading, and Section 8 summarizes the study and outlines future work.

## 2. P2P Energy Trading Model

**Figure 1:**
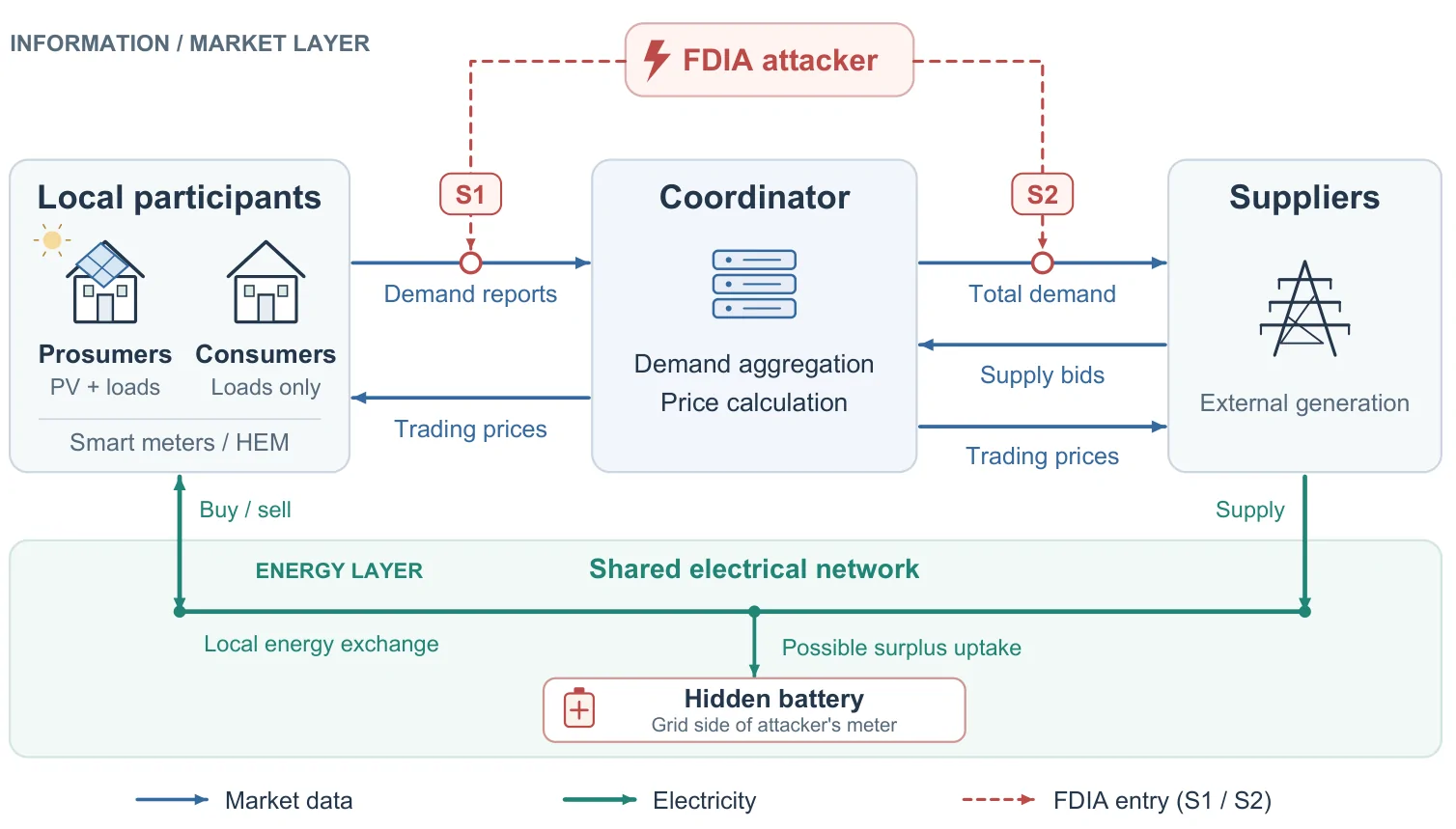
Conceptual P2P energy trading model. Blue arrows denote market messages and green links denote electricity exchange. Red dashed arrows mark FDIA entry points: individual demand reports (S1) and aggregated demand (S2). The hidden battery represents possible unmetered surplus uptake; the coordinator processes information rather than physical power.

We consider a community-based P2P energy trading model with four types of agents (see Figure 1): *M* prosumer agents consisting of pure consumers and prosumers with solar generation; *N* supplier agents generating energy from solar, wind, or conventional power plants; one coordinator agent connecting suppliers; and one attacker agent manipulating data.

For each hour, prosumers determine how much energy to buy or sell based on their solar generation and own consumption, and send their values to the coordinator. The attacker intercepts these demand data and falsifies them before they reach the coordinator. The coordinator then aggregates all reported demand, including the falsified entries, to calculate the net load and forwards it to the suppliers. Suppliers, who produce their own energy and sell it to prosumers whenever local solar generation is insufficient, respond with bids based on this net load. The coordinator uses these bids to calculate trading prices for the local market and distributes them to all agents. Both suppliers and prosumers then update their bids according to the new prices, and the process repeats.

At each iteration, the attacker inflates the falsified prosumer demand so that the market procures more energy than the true demand requires, and then freely consumes this surplus energy using a hidden battery. This iterative process continues until the external price converges.

## 3. Attack Scenarios

In general, an FDIA introduces false or manipulated data into a power system. Such attacks can originate from outside the market, where an adversary or third-party attacker manipulates information exchanged among market participants (e.g., prosumers, consumers, and suppliers), such as bids or price offers, in order to cause financial loss to participants or disrupt the market process [37]. Alternatively, the adversary can be one of the market participants itself, such as a malicious prosumer, who directly injects false data into the market system or attacks through devices (e.g., a smart meter or HEM unit) to gain their own financial benefit. In either case, the market operator (the coordinator) ends up making decisions based on false information that does not match the true market scenario. In this section, we design two threat scenarios in which FDIA adversely changes demand data [37].

### 3.1. Threat Scenario 1

In this threat scenario, the attacker is a malicious energy seller, such as a supplier, who poses as a prosumer in the market. This attack can be motivated in two ways: the attacker may act on behalf of a supplier to directly profit from the attack, or the attacker may aim to benefit suppliers indirectly by reducing the incentive for consumers to become energy-selling prosumers. In either case, the attacker attempts to find the best way to modify prosumers’/consumers’ demands so as to gain benefit or cause sabotage, while minimizing the chance of being detected.

Here, we assume that each prosumer’s demand is falsified as a function of the prosumer’s own true demand. The true demand and falsified demand of prosumer *i* are denoted by $d_{i}$ and $d_{i}^{\prime }$, respectively. Then, the falsified demand $d_{i}^{\prime }$ can be represented by $d_{i}^{\prime }=d_{i}\left(1+f\left(d_{i}\right)\right)$, where f(·) is some function. At this stage, the amount of supplied electricity no longer matches the prosumer’s actual total demand, generally resulting in a power grid imbalance. Specifically, if the supply side delivers more power than is actually required, the surplus is not consumed by the prosumer; this is because the targeted prosumer is unaware that their reported demand has been artificially inflated by the attacker. Consequently, the attacker gains the opportunity to consume this surplus power for free. One such method involves installing a battery storage system on the “grid side” of the smart meter located at the attacker’s premises. Since the battery is installed on the grid side, the power drawn into it is not recorded by the smart meter, allowing the attacker to obtain that electricity at no cost.

### 3.2. Threat Scenario 2

Similarly to Threat Scenario 1, this scenario envisions attacks aimed at sabotage or the unauthorized acquisition of energy. However, since individually attacking the Home Energy Management (HEM) systems of numerous prosumers is difficult, we present an alternative scenario in which energy can be obtained for free without targeting individual HEM systems.

In this threat scenario, an attacker manipulates the total demand calculated by the coordinator. Consequently, all prices are altered based on the total demand tampered with by the attacker. Let $D_{i}$ and $D_{i}^{\prime }$ represent the true total demand and the tampered total demand, respectively. In this case, the tampered demand $D_{i}^{\prime }$ can be expressed as $D_{i}^{\prime }=D_{i}\left(1+F\left(D_{i}\right)\right)$, where F(·) is a certain function.

In this attack, the adversary operates at the network level, targeting the communication between the coordinator and the suppliers. The attacker alters the total demand calculated by the coordinator; since the coordinator determines both the external and internal prices based on this total demand, the falsified demand causes both prices to be distorted accordingly. The attacker repeats this demand manipulation at every iteration until this iteration is finished. At the end of the iteration, the suppliers provide more energy than the demand required in the real situation. As a result, a grid imbalance occurs. A hidden battery may absorb surplus energy and conceal a balance deviation, depending on its physical placement and control. The detector focuses on measurement integrity; battery state and physical power-flow dynamics lie outside its input representation.

### 3.3. Scope of the Detection Task

The trading process and attack descriptions define the application context. The detector maps the released measurement features to an attack-class probability. It does not estimate market profit or battery state. The demand–generation–consumption residual is treated as a physical relation rather than a replacement for the reference attack label. The original labels and declared worksheet define the classification task.

## 4. SBOA-Inspired PSO–XGBoost Detection Framework

### 4.1. Scenario-Specific Classification Inputs

Let $D_{\mathrm{tr}}={\left\{\left(x_{i},y_{i}\right)\right\}}_{i=1}^{n_{\mathrm{tr}}}$ and $D_{\mathrm{te}}$ denote the released training and test partitions, respectively. The attack class is y=1 and the normal class is y=0. Original workbook values 2 and 1 are mapped to these internal labels without changing their meaning. A separate classifier is specified for each household-group/threat-scenario combination.

The input dimension depends on the threat scenario. Scenario 1 uses generation, demand, shiftable load, base load, and consumption ($d_{x}=5$). Scenario 2 uses total demand, total consumption, and total generation ($d_{x}=3$). Household identifiers, timestamps, row numbers and hand-crafted balance residuals are excluded from both feature sets. Column names that differ between released worksheets are mapped to these scenario-specific physical quantities. The resulting scenario-dependent feature mapping is $f_{\theta }:R^{d_{x}}\to \left[0,1\right]$.

For each sample, the declared decision rule is

(1) $${\hat{y}}_{i}=I\left\{f_{\theta }\left(x_{i}\right)\geq 0.5\right\}.$$

The threshold is fixed in advance. Any threshold optimization must be restricted to training/validation data and counted as part of model selection.

### 4.2. Classifiers and the XGBoost Search Space

The comparison includes support vector machines (SVM), Gaussian naive Bayes (NB), decision trees (DT), *k*-nearest neighbors (KNN), random forests (RF), multilayer perceptrons (MLP), and fixed-parameter XGBoost [25,26,28,29,31,33,35]. These names identify model families; they do not by themselves determine a confusion matrix. For methods requiring scaling, a standardizer must be fitted on the training portion of each fold, then applied to that fold’s validation portion. The final standardizer is fitted on the full training partition only.

XGBoost combines regression trees through a regularized boosting objective. For additive tree $h_{k}$, a representative objective is

(2) $$L^{\left(k\right)}=\sum_{i=1}^{n_{\mathrm{tr}}}\ell \left(y_{i},{\hat{z}}_{i}^{\left(k-1\right)}+h_{k}\left(x_{i}\right)\right)+\Omega \left(h_{k}\right),$$

where ${\hat{z}}_{i}$ denotes the current prediction score, *ℓ* is the training loss and Ω penalizes tree complexity. The optimizer searches hyperparameters rather than sample labels, input features, or test predictions.

Each particle has a normalized position $z_{i}\in {\left[0,1\right]}^{8}$. The decoder maps this position to the ranges in Table 1, rounding tree count and depth to integers. Logarithmic coordinates are used for the learning rate and regularization coefficients. Here the particle dimension is $d_{\theta }=8$, distinct from the input-feature dimension $d_{x}$.

### 4.3. Training-Only Fitness and Candidate Confirmation

Both optimizers maximize the same three-fold validation score. With metrics expressed as fractions in [0,1], define

(3) $$J\left(\theta \right)=\frac{1}{3}\sum_{k=1}^{3}\frac{A_{k}\left(\theta \right)+P_{k}\left(\theta \right)+R_{k}\left(\theta \right)+F_{1,k}\left(\theta \right)}{4}.$$

The folds are stratified partitions of $D_{\mathrm{tr}}$ and are held fixed within a search. A zero-division precision or F1 is assigned zero. Equation (3) is a declared composite objective, not four independent optimization problems: its components are correlated and it is not asserted to be the best objective for every deployment. F1 remains the principal reporting metric for the gain analysis. An F1-only or cost-sensitive objective would constitute a separately specified sensitivity experiment.

Candidate confirmation uses a second stratified three-fold partition of the same training data. The fixed XGBoost reference and the three highest-scoring distinct non-reference parameter configurations are re-evaluated. Their selection score is the average of the search-fold and confirmation-fold scores. The highest-scoring candidate is fitted once on all training samples and evaluated on the untouched test partition. Confirmation is an additional training-data selection step, not an independent estimate of test performance. Ties retain the earlier candidate, and repeated evaluations still consume the declared budget.

### 4.4. PSO Reference Update

For particle *i* at search round *t*, let $p_{i}$ denote its personal best position and g the global best. A standard bounded PSO update [41] is

(4) $$\begin{array}{cc} v_{i}^{t+1} & ={\mathrm{clip}}_{\left[-v_{\max},v_{\max}\right]}\left(wv_{i}^{t}+c_{1}r_{1,i}⊙\left(p_{i}-z_{i}^{t}\right)+c_{2}r_{2,i}⊙\left(g-z_{i}^{t}\right)\right), \end{array}$$

(5) $$\begin{array}{cc} {\tilde{z}}_{i}^{t+1} & =\Pi_{{\left[0,1\right]}^{8}}\left(z_{i}^{t}+v_{i}^{t+1}\right), \end{array}$$

where ⊙ is elementwise multiplication, the entries of $r_{1,i}$ and $r_{2,i}$ are independent uniform random variables, and Π clips a candidate to the normalized box. The reference design sets w=0.7, $c_{1}=c_{2}=1.5$, and $v_{\max}=0.2$. Initial velocities lie in ${\left[-0.1,0.1\right]}^{8}$. The swarm includes the encoded fixed XGBoost reference as its first particle and samples the remaining positions uniformly. These settings also define the PSO component of the hybrid, so that the proposed difference is the candidate-proposal rule.

### 4.5. Three Hunting-Inspired Hybrid Proposals

The local hybrid implementation motivates the following explicit proposal definition, adapted from the hunting operators of SBOA [39]. Let *G* be the number of evaluation rounds including initialization, and let q=t/(G−1) for a noninitial round t=1,…,G−1. The progress-dependent candidate is

(6) $$h_{i}^{t}=\left\{\begin{array}{cc} z_{i}^{t}+u⊙\left(z_{a}^{t}-z_{b}^{t}\right), & q<1/3,\\ g+\exp\left(q^{4}\right)\left(\eta -0.51\right)⊙\left(g-z_{i}^{t}\right), & 1/3\leq q<2/3,\\ g+{\left(1-q\right)}^{2q}z_{i}^{t}⊙L, & 2/3\leq q\leq 1. \end{array}\right.$$

Indices *a* and *b* are distinct and exclude *i*; u is uniform on ${\left[0,1\right]}^{8}$ and η has independent standard-normal entries. The first proposal uses differences between swarm positions, the second perturbs the current global best at a scale depending on the particle, and the third uses a shrinking heavy-tailed perturbation. They are adaptations within PSO, not a claim of a new original SBOA formulation.

For the third proposal, each component of L is generated as

(7) $$\begin{array}{cc} L_{j} & =\frac{0.5\;U_{j}}{\max(|V_{j}{\left|,10^{-12}\right)}^{1/\beta }},\;U_{j}∼N\left(0,\sigma_{\beta }^{2}\right),\;V_{j}∼N\left(0,1\right),\;\beta =1.5, \end{array}$$

(8) $$\begin{array}{cc} \sigma_{\beta } & ={\left[\frac{\Gamma (1+\beta )\sin(\pi \beta /2)}{\Gamma \left(\left(1+\beta \right)/2\right)\beta \;2^{(\beta -1)/2}}\right]}^{1/\beta }. \end{array}$$

The distributional expression and the numerical floor specify how proposals are generated. They do not imply that the resulting classifier has a heavy-tailed error distribution.

A fixed fraction ρ=0.5 of the particles receives a hybrid proposal instead of the PSO position proposal at each noninitial round. With zero-based particle indices, select *i* when (i+t)modN<round(Nρ), which rotates the selected subset. Each candidate is clipped to ${\left[0,1\right]}^{8}$ and evaluated once. Hybrid proposals replace the current particle only if their score improves by more than $10^{-12}$; accepted hybrid proposals reset that particle’s velocity to zero. A PSO proposal follows the ordinary position update even when its score is lower, while personal/global bests are retained separately. Candidates for a round are generated from the pre-evaluation swarm. These rules preserve one scored candidate per particle per round.

At q=1, the last coefficient in Equation (6) is zero, so the selected proposals coincide with the current best. These repeated candidates are charged to the budget, rather than described as new exploratory evaluations. The specified hybrid omits SBOA’s separate escape phase and does not use the older NDIW/ALVLF schedules. Its possible benefit is an empirical question; no global-convergence guarantee is claimed.

Finally, we summarize the proposed algorithm as Algorithm 1.**Algorithm 1** Specified SBOA-inspired PSO search for an XGBoost detector**Require:** Training partition; decoder; *N*, *G*, ρ; two training-only CV partitions **Ensure:** Selected hyperparameters and one final XGBoost model
  1:Initialize normalized particles, velocities and personal/global best scores  2:Include the fixed XGBoost reference as the first particle  3:**for** t=0 to G−1 **do**  4:   **if** t=0 **then**  5:       Use initial particle positions as candidates  6:   **else**  7:       Compute bounded PSO proposals using Equations (4) and (5)  8:       Replace proposals for the rotating subset using Equation (6)  9:   **end if**10:   **for** each particle’s candidate **do**11:       Decode and evaluate the candidate using Equation (3)12:       Update personal and global bests if the score improves13:       Apply the PSO or greedy hybrid acceptance rule14:   **end for**15:**end for**16:Confirm the reference and the best three distinct non-reference candidates17:Select by the average search/confirmation score18:Fit the selected configuration on the full training partition19:Predict the untouched test partition once under the fixed threshold rule


### 4.6. Workflow and Computational Accounting

The search budget is N=12, G=12, yielding 144 scored candidates including initialization. Four candidate confirmations add 12 fold fits, so (144+4)×3=444 training-fold fits are allocated to each optimizer, followed by one final fit. If $C_{\mathrm{fit}}$ denotes an average fold-fit cost, the dominant training work scales as $O(\left(NG+4\right)3C_{\mathrm{fit}})$, while vector updates scale as $O(NGd_{\theta })$. Equal fit counts can still produce different total times because candidate configurations differ in tree count and depth.

## 5. Evaluation Protocol

### 5.1. Dataset Provenance and Four Evaluation Settings

The Austin attack dataset combines household energy measurements with author-defined attack scenarios [37,38]. The evaluation retains the released training/test separation and original attack labels. Group 1 comprises 20 solar-equipped prosumers and 30 consumers; Group 2 comprises 13 prosumers, 10 consumers, an attacker and three suppliers. Table 2 lists the workbook row counts for the four household-group/threat-scenario combinations.

The source manuscript assigns the 1 August 2018 data to training and the 1 July 2018 data to testing. The released file assignments are retained. Generalization to later periods requires a separate chronological evaluation.

### 5.2. Reference Settings and Selection Boundaries

The search space and optimization objective are specified in Section 4. The fixed baseline configurations are an RBF SVM with C=1 and scale-based kernel width, Gaussian NB with smoothing $10^{-9}$, a Gini DT with depth 5 and minimum leaf size 2, and a five-neighbor distance-based KNN. RF uses 100 trees and depth 5. The MLP uses one two-unit tanh hidden layer, L-BFGS and $L_{2}$ coefficient 1. The fixed XGBoost reference uses 100 trees, depth 3, learning rate 0.1, row/column sampling of 1, minimum child weight of 1, α=0.001 and λ=1.

The fixed RF and MLP configurations provide controls with a defined model capacity. The comparison therefore concerns these reference settings rather than the best achievable performance of every classifier family. Budget-matched tuning of the baseline learners and a random-search control are useful extensions for assessing optimizer-specific gains.

### 5.3. Evaluation Metrics

For attacks as the positive class, let TP, FP, FN, and TN denote true positives, false positives, false negatives, and true negatives. The four primary metrics, reported in percent, are accuracy (*A*), precision (*P*), recall (*R*) and F1 ($F_{1}$):

(9) $$\begin{array}{cc} A & =100\frac{TP+TN}{TP+FP+FN+TN}, \end{array}$$

(10) $$\begin{array}{cc} P & =100\frac{TP}{TP+FP}, \end{array}$$

(11) $$\begin{array}{cc} R & =100\frac{TP}{TP+FN}, \end{array}$$

(12) $$\begin{array}{cc} F_{1} & =100\frac{2TP}{2TP+FP+FN}. \end{array}$$

The fold scores in Equation (3) use these metrics divided by 100. The normal-class false-positive rate is FPR=100FP/(FP+TN). Precision and specificity (100−FPR, in percent) are not interchangeable: their denominators differ. Equal-scenario averages are calculated by averaging each metric over the four settings, rather than pooling their samples. In particular, the reported mean F1 is the mean of four F1 values, not the harmonic mean of the averaged precision and recall.

All four metrics are calculated from the same confusion matrix for each method and setting. The positive and negative class totals are fixed within each test set, enabling direct comparison of missed attacks and false alarms. Appendix A provides the complete matrices for numerical verification.

### 5.4. Time Definitions

Search time includes cross-validation fitting, candidate selection, confirmation, and optimizer work. Final-training time refers to one refit on all training samples. Prediction time refers to the complete test batch. Preprocessing required for each stage is included, while file reading, report generation and plot rendering are excluded from this declared timing boundary. The total is

(13) $$t_{\mathrm{total}}=t_{\mathrm{search}}+t_{\mathrm{fit}}+10^{-3}t_{\mathrm{pred},\mathrm{ms}}.$$

All time terms are in seconds except $t_{\mathrm{pred},\mathrm{ms}}$, which is in milliseconds. A zero search time for a fixed baseline indicates that the timing budget includes no hyperparameter search for that method. Dividing batch-prediction time by the test-set size gives amortized time per sample; it is a throughput measure rather than an individual-request latency guarantee.

## 6. Results and Analysis

### 6.1. Detection Performance in Each Setting

Table 3 and Table 4 summarize accuracy, precision, recall and F1 for the four settings, with the two household groups placed side by side. Fixed XGBoost provides a stronger reference than the other fixed configurations, PSO tuning improves its detection performance, and the hybrid search adds a further gain. The corresponding error counts are reported in Appendix A.

**Figure 2:**
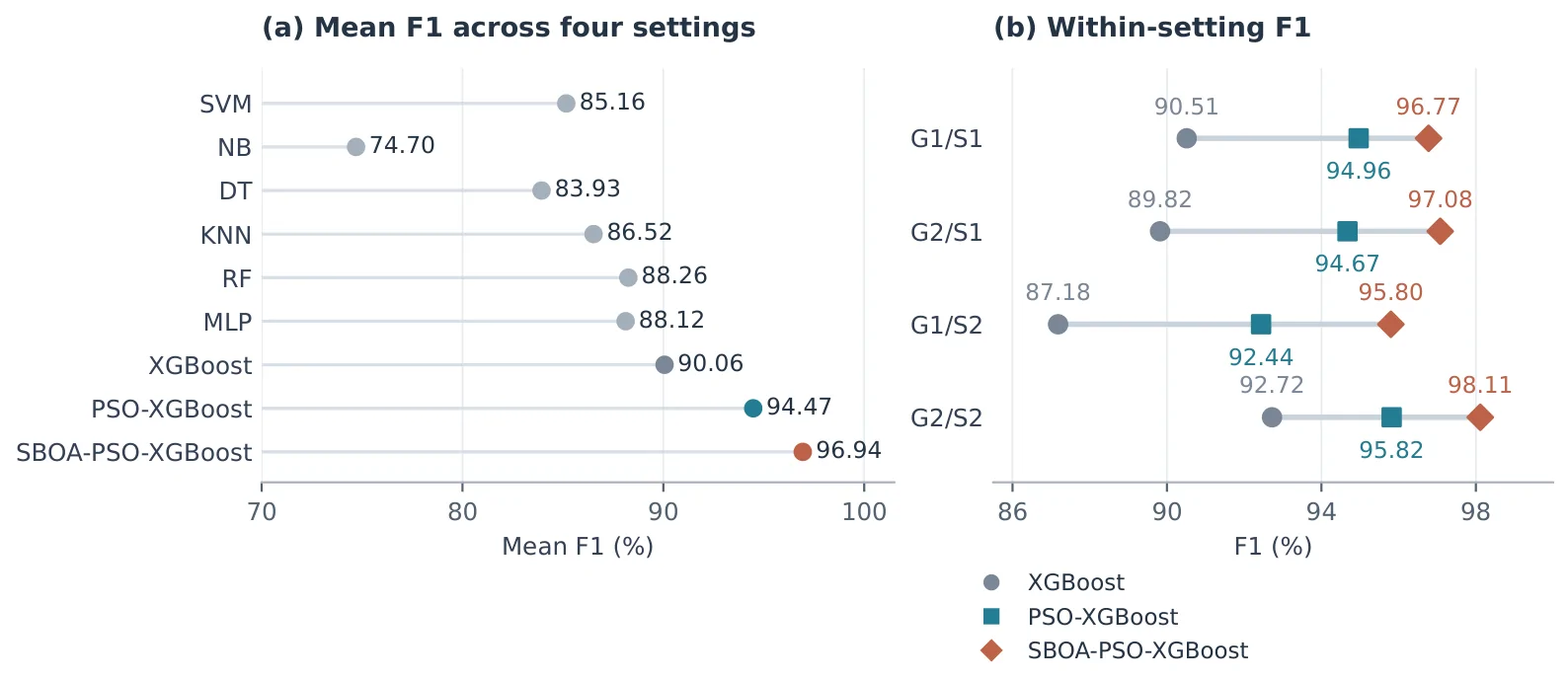
F1 comparison: (**a**) equal-scenario means for all methods; (**b**) within-setting contrasts for the three XGBoost variants. Points are annotated with their values. Both axes are expanded for readability; connecting segments indicate comparisons, not learning trajectories.

In Threat Scenario 1, the F1 score progresses from 90.51% to 94.96% and 96.77% in Group 1, and from 89.82% to 94.67% and 97.08% in Group 2. The hybrid therefore adds 1.81 and 2.40 percentage points over PSO. The two stages of improvement are also visible in Figure 2b.

In Threat Scenario 2, the hybrid adds 3.36 percentage points over PSO in Group 1 and 2.30 points in Group 2. G1/S2 remains the most challenging setting, with F1 of 95.80%, two false positives and three false negatives. In G2/S2, attacks account for 133 of 178 test samples. The hybrid F1 of 98.11% can therefore coexist with accuracy of 97.19%; two false alarms among only 45 normal samples still yield a 4.44% false-positive rate. Attack-class F1 alone does not describe normal-class errors.

The G1/S1 hybrid matrix is (TP,FP,FN,TN)=(135,4,5,506), giving precision 135/139=97.12%, recall 135/140=96.43%, and F1 270/279=96.77%. Their distinction follows from unequal false-positive and false-negative counts. Equality would also be mathematically valid if FP=FN and TP>0.

### 6.2. Equal-Scenario Summary

Figure 2a summarizes the equal-scenario mean F1 for all nine methods. The three XGBoost variants reach 90.06%, 94.47%, and 96.94%, corresponding to increases of 4.42 and 2.47 percentage points. Differences are calculated before rounding; percentage-point gains are not relative percentage increases. The hybrid’s mean accuracy, precision and recall are 98.12%, 97.18% and 96.70%. Each setting receives equal weight in these averages.

### 6.3. Computational Cost

**Figure 3:**
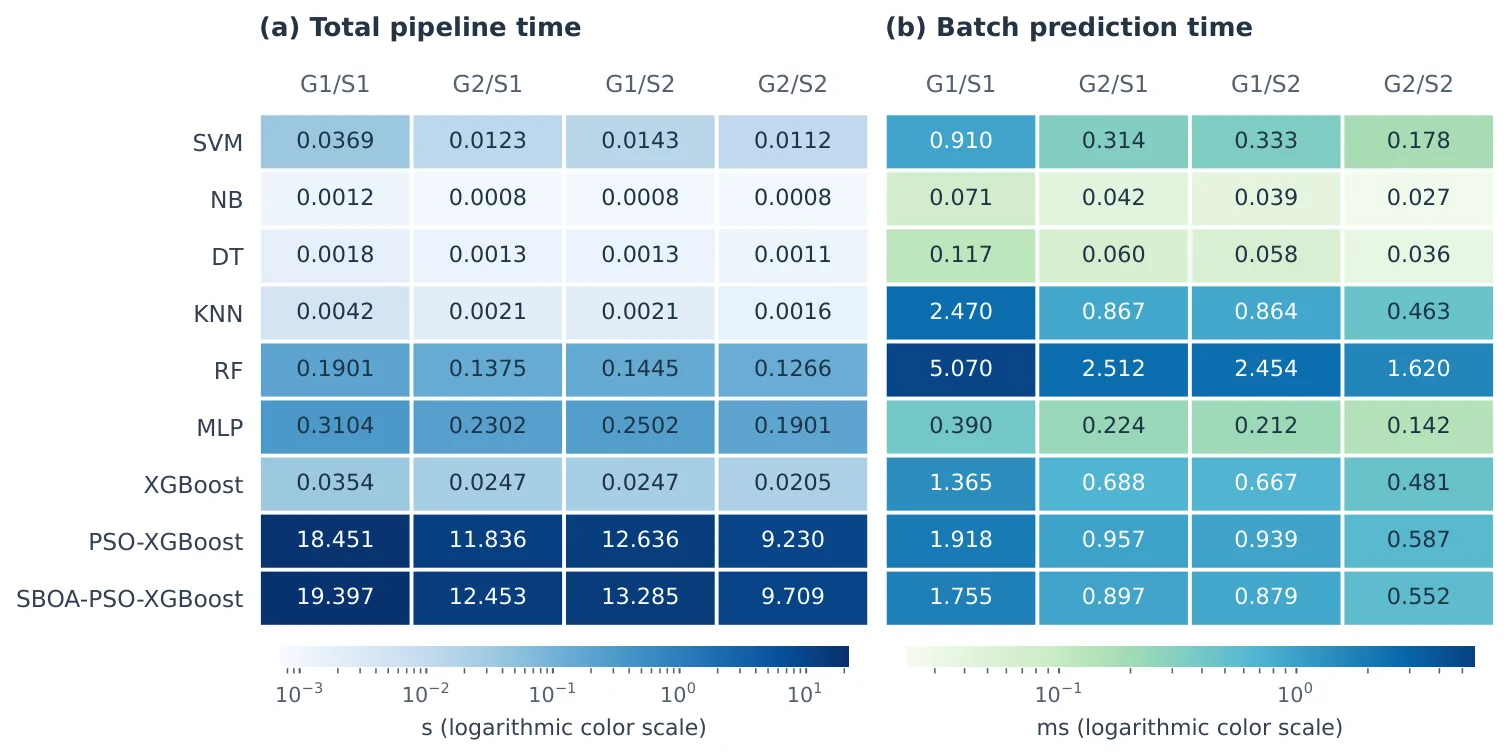
Computational cost across the four settings: (**a**) search + final fit + batch prediction, in seconds; (**b**) prediction for the complete test batch, in milliseconds. Columns in (**b**) contain 650, 299, 303 and 178 samples, respectively. The two color scales have different units and ranges.

Figure 3 compares total pipeline and batch-prediction times across all methods and settings. Cell labels give numerical values; logarithmic color scales show the range of costs.

The optimized methods incur repeated fitting during search, whereas the deployed detector remains a single XGBoost classifier. Offline optimization cost is therefore separated from prediction cost. The hybrid’s batch-prediction times remain close to PSO-XGBoost’s, while NB and DT have lower absolute costs. This distinction is relevant when selecting a detector under training and inference constraints.

Across all settings, total time is 52.152 s for PSO and 54.843 s for the hybrid, an increase of 5.16%. Table 5 pairs that modest additional cost with F1 gains of 1.81–3.36 percentage points. The ratio does not measure computational complexity or isolate the cost of SBOA arithmetic: different candidate hyperparameters can incur different fitting costs. Communication, batching, market clearing and mitigation remain outside this timing boundary.

## 7. Discussion and Research Contributions

### 7.1. Contribution of the Hybrid Optimization Framework

The methodological contribution lies in the specified integration of three SBOA-inspired hunting operators with PSO’s personal-best and global-best memories. A rotating subset of particles receives progress-dependent hunting proposals, with greedy acceptance and velocity reset after an accepted proposal, while the remaining particles follow the PSO update. This combination defines how the two search mechanisms cooperate within a single population. The common parameter encoding, training-only validation objective and candidate-evaluation budget make the proposal mechanism a directly comparable component of the optimization framework.

The comparison of fixed XGBoost, PSO-XGBoost and SBOA-PSO-XGBoost distinguishes parameter tuning from the additional hybrid search mechanism. Together with explicit candidate confirmation and scenario-specific feature definitions, this comparison structure connects the optimizer design to the FDIA detection task and provides a basis for investigating the contribution of individual search operators.

### 7.2. Detection Performance and Computational Efficiency

The four-setting comparison shows an increase in mean F1 of 4.42 percentage points from fixed XGBoost to PSO-XGBoost and a further increase of 2.47 points with the hybrid. Both false positives and false negatives decrease in each setting, indicating that the improvement combines attack detection with false-alarm control at the fixed decision threshold.

The computation-time comparison complements this detection analysis. Relative to PSO-XGBoost, the hybrid increases total time by 5.16% across the four settings while retaining similar batch-prediction cost. Parameter search is performed offline, and the selected configuration is deployed as one XGBoost classifier. This separation places the additional search effort in model preparation and makes the detection–cost relationship explicit for subsequent deployment planning.

### 7.3. Significance for P2P Energy Trading

The framework addresses the integrity of individual demand reports and coordinator-level aggregate measurements through separate scenario-specific classifiers. Using physical energy quantities and excluding User ID bases the predictive representation on measured trading conditions. The two scenario definitions also connect the detection task to the information-exchange points shown in Figure 1.

In a P2P market workflow, such a classifier could screen incoming measurements before they are used in aggregation or market clearing. Flagged reports could then be checked against supporting measurements or referred for further verification. Separating offline optimization from online classification provides an architecture in which the coordinator can receive detection decisions without repeating the swarm search for each new report.

## 8. Conclusions and Future Work

This paper presents an SBOA-inspired PSO framework for tuning an XGBoost detector of false data injection attacks in P2P energy trading. Three hunting-inspired operators are integrated into the PSO candidate-generation process, with a common search space, training-only validation objective and candidate budget. The study organizes nine classifiers across four settings, combining scenario-specific physical inputs with classification metrics, confusion matrices and computational-cost comparisons.

Fixed XGBoost, PSO-XGBoost and SBOA-PSO-XGBoost achieve equal-setting mean F1 scores of 90.06%, 94.47% and 96.94%, respectively. The hybrid’s 2.47-percentage-point gain over PSO is accompanied by a 5.16% increase in total computation time and similar batch-prediction cost. The comparison highlights the contribution of offline parameter search while preserving a single classifier for inference.

Future work will prioritize cross-household, cross-season and cross-dataset validation, together with feature-importance analysis to examine the basis of model decisions. The attack-generation process and its physical constraints will be refined, and hunting-operator ablations will be conducted alongside comparisons with grid search, random search, Bayesian optimization, GA-XGBoost and GWO-XGBoost under the same evaluation budget. Repeated experiments, statistical tests and measured runtimes will further assess detection performance, convergence behavior and computational cost. Robustness to measurement noise, missing data and adaptive attacks will also be evaluated, while attacker localization and the integration of detection into market operations will be explored.

## Figures and Tables

**Table 1:** Common hyperparameter search space for PSO-XGBoost and SBOA-PSO-XGBoost.

Parameter	Range	Encoding
Number of trees	20–320	Rounded integer
Maximum tree depth	1–8	Rounded integer
Learning rate	0.01–0.30	$\log_{10}$ scale
Row subsampling	0.65–1.00	Linear
Column subsampling	0.65–1.00	Linear
Minimum child weight	0.5–10	Linear
$L_{1}$ coefficient α	$10^{-4}$–10	$\log_{10}$ scale
$L_{2}$ coefficient λ	0.01–20	$\log_{10}$ scale

**Table 2:** Training and test sample counts, test-class composition and feature dimensions.

Setting	Train	Test	Attacks	Normal	Features
G1/S1	650	650	140	510	5
G2/S1	299	299	85	214	5
G1/S2	303	303	60	243	3
G2/S2	200	178	133	45	3

**Table 3:** Detection performance for Threat Scenario 1. All entries are percentages; bold type marks the hybrid design.

Method	G1/S1				G2/S1			
	Accuracy	Precision	Recall	F1	Accuracy	Precision	Recall	F1
SVM	93.85	88.46	82.14	85.19	91.64	87.50	82.35	84.85
NB	89.38	79.83	67.86	73.36	86.29	78.21	71.76	74.85
DT	93.38	85.93	82.86	84.36	90.97	84.52	83.53	84.02
KNN	94.46	89.39	84.29	86.76	92.31	87.80	84.71	86.23
RF	95.38	91.04	87.14	89.05	93.31	90.12	85.88	87.95
MLP	95.23	91.60	85.71	88.56	92.98	88.10	87.06	87.57
XGBoost	96.00	92.54	88.57	90.51	94.31	91.46	88.24	89.82
PSO-XGBoost	97.85	95.65	94.29	94.96	96.99	95.24	94.12	94.67
**SBOA-PSO-XGBoost**	**98.62**	**97.12**	**96.43**	**96.77**	**98.33**	**96.51**	**97.65**	**97.08**

**Table 4:** Detection performance for Threat Scenario 2. All entries are percentages; bold type marks the hybrid design.

Method	G1/S2				G2/S2			
	Accuracy	Precision	Recall	F1	Accuracy	Precision	Recall	F1
SVM	92.74	85.19	76.67	80.70	85.39	92.80	87.22	89.92
NB	87.79	72.55	61.67	66.67	76.97	87.70	80.45	83.92
DT	91.75	80.70	76.67	78.63	83.71	91.94	85.71	88.72
KNN	93.40	85.71	80.00	82.76	85.96	92.86	87.97	90.35
RF	94.06	87.50	81.67	84.48	87.64	93.70	89.47	91.54
MLP	94.06	86.21	83.33	84.75	87.64	93.02	90.23	91.60
XGBoost	95.05	89.47	85.00	87.18	89.33	94.53	90.98	92.72
PSO-XGBoost	97.03	93.22	91.67	92.44	93.82	96.92	94.74	95.82
**SBOA-PSO-XGBoost**	**98.35**	**96.61**	**95.00**	**95.80**	**97.19**	**98.48**	**97.74**	**98.11**

**Table 5:** Incremental quality–cost trade-off: hybrid relative to PSO. F1 gain is in percentage points (pp).

Setting	PSO (s)	Hybrid (s)	Extra Time (%)	F1 Gain (pp)
G1/S1	18.451	19.397	5.13	1.81
G2/S1	11.836	12.453	5.21	2.40
G1/S2	12.636	13.285	5.14	3.36
G2/S2	9.230	9.709	5.19	2.30

## Data Availability

The data analyzed in this study are described in the manuscript.

## Appendix A. Confusion Matrices

Table A1 presents the 36 confusion matrices for the four settings and nine methods. Counts within each setting have identical positive and negative totals.

**Table A1:** Confusion matrices for all methods and settings. Columns within each setting are TP, FP, FN and TN.

Method	G1/S1				G2/S1				G1/S2				G2/S2			
	TP	FP	FN	TN	TP	FP	FN	TN	TP	FP	FN	TN	TP	FP	FN	TN
SVM	115	15	25	495	70	10	15	204	46	8	14	235	116	9	17	36
NB	95	24	45	486	61	17	24	197	37	14	23	229	107	15	26	30
DT	116	19	24	491	71	13	14	201	46	11	14	232	114	10	19	35
KNN	118	14	22	496	72	10	13	204	48	8	12	235	117	9	16	36
RF	122	12	18	498	73	8	12	206	49	7	11	236	119	8	14	37
MLP	120	11	20	499	74	10	11	204	50	8	10	235	120	9	13	36
XGBoost	124	10	16	500	75	7	10	207	51	6	9	237	121	7	12	38
PSO-XGBoost	132	6	8	504	80	4	5	210	55	4	5	239	126	4	7	41
**SBOA-PSO-XGBoost**	**135**	**4**	**5**	**506**	**83**	**3**	**2**	**211**	**57**	**2**	**3**	**241**	**130**	**2**	**3**	**43**

## References

[1] Fang X., Misra S., Xue G., Yang D. (2011). Smart grid—The new and improved power grid: A survey. IEEE Commun. Surv. Tutor..

[2] Ohanu C.P., Rufai S.A., Oluchi U.C. (2024). A comprehensive review of recent developments in smart grid through renewable energy resources integration. Heliyon.

[3] khan S., Mazhar T., Shahzad T., khan M.A., Rehman A.U., Hamam H. (2025). Integration of smart grid with industry 5.0: Applications, challenges and solutions. Meas. Energy.

[4] Depuru S.S.S.R., Wang L., Devabhaktuni V., Gudi N. (2011). Smart meters for power grid—Challenges, issues, advantages and status. Proceedings of the 2011 IEEE/PES Power Systems Conference and Exposition.

[5] Zheng J., Gao D.W., Lin L. (2013). Smart meters in smart grid: An overview. Proceedings of the 2013 IEEE Green Technologies Conference (GreenTech).

[6] Raza M.H., Rind Y.M., Javed I., Zubair M., Mehmood M.Q., Massoud Y. (2023). Smart meters for smart energy: A review of business intelligence applications. IEEE Access.

[7] Díaz Redondo R.P., Fernández-Vilas A., Fernández dos Reis G. (2020). Security Aspects in Smart Meters: Analysis and Prevention. Sensors.

[8] Munoz O., Ruelas A., Rosales P., Acuña A., Suastegui A., Lara F. (2022). Design and development of an IoT smart meter with load control for home energy management systems. Sensors.

[9] Rind Y.M., Raza M.H., Zubair M., Mehmood M.Q., Massoud Y. (2023). Smart energy meters for smart grids, an internet of things perspective. Energies.

[10] Patrizi G., Garzon Alfonso C., Calandroni L., Bartolini A., Iturrino Garcia C., Paolucci L., Grasso F., Ciani L. (2024). Anomaly detection for power quality analysis using smart metering systems. Sensors.

[11] Zhang B., Lin G., Zheng K., Du J. (2026). Anomaly Detection and Data Repair for Smart Meter Data in Smart Cities: A Comprehensive Review and Future Perspectives. Sensors.

[12] Siano P. (2014). Demand response and smart grids—A survey. Renew. Sustain. Energy Rev..

[13] Stanelyte D., Radziukyniene N., Radziukynas V. (2022). Overview of demand-response services: A review. Energies.

[14] Ranaboldo M., Aragüés-Peñalba M., Arica E., Bade A., Bullich-Massagué E., Burgio A., Caccamo C., Caprara A., Cimmino D., Domenech B. (2024). A comprehensive overview of industrial demand response status in Europe. Renew. Sustain. Energy Rev..

[15] Zhang C., Wu J., Long C., Cheng M. (2017). Review of Existing Peer-to-Peer Energy Trading Projects. Energy Procedia.

[16] Zhang C., Wu J., Zhou Y., Cheng M., Long C. (2018). Peer-to-peer energy trading in a microgrid. Appl. Energy.

[17] Tushar W., Saha T.K., Yuen C., Smith D., Poor H.V. (2020). Peer-to-peer trading in electricity networks: An overview. IEEE Trans. Smart Grid.

[18] Soto E.A., Bosman L.B., Wollega E., Leon-Salas W.D. (2021). Peer-to-peer energy trading: A review of the literature. Appl. Energy.

[19] Liang G., Zhao J., Luo F., Weller S.R., Dong Z.Y. (2016). A review of false data injection attacks against modern power systems. IEEE Trans. Smart Grid.

[20] Wang Q., Tai W., Tang Y., Ni M. (2019). Review of the false data injection attack against the cyber-physical power system. IET Cyber-Phys. Syst. Theory Appl..

[21] Musleh A.S., Chen G., Dong Z.Y. (2019). A survey on the detection algorithms for false data injection attacks in smart grids. IEEE Trans. Smart Grid.

[22] Ahmed M., Pathan A.S.K. (2020). False data injection attack (FDIA): An overview and new metrics for fair evaluation of its countermeasure. Complex Adapt. Syst. Model..

[23] Elsisi M., Mahmoud K., Lehtonen M., Darwish M.M. (2021). Reliable industry 4.0 based on machine learning and IOT for analyzing, monitoring, and securing smart meters. Sensors.

[24] Hernández Á., Nieto R., de Diego-Otón L., Pérez-Rubio M.C., Villadangos-Carrizo J.M., Pizarro D., Ureña J. (2024). Detection of anomalies in daily activities using data from smart meters. Sensors.

[25] Webb G.I., Keogh E., Miikkulainen R. (2010). Naïve Bayes. Encycl. Mach. Learn..

[26] Kotsiantis S.B. (2013). Decision trees: A recent overview. Artif. Intell. Rev..

[27] Song Y.-y., Lu Y. (2015). Decision tree methods: Applications for classification and prediction. Shanghai Arch. Psychiatry.

[28] Peterson L.E. (2009). K-nearest neighbor. Scholarpedia.

[29] Wang L. (2005). Support Vector Machines: Theory and Applications.

[30] Suthaharan S. (2016). Support vector machine. Machine Learning Models and Algorithms for Big Data Classification: Thinking with Examples for Effective Learning.

[31] Cutler A., Cutler D.R., Stevens J.R. (2012). Random forests. Ensemble Machine Learning.

[32] Salman H.A., Kalakech A., Steiti A. (2024). Random forest algorithm overview. Babylon. J. Mach. Learn..

[33] Delashmit W.H., Manry M.T. Recent developments in multilayer perceptron neural networks. Proceedings of the Seventh Annual Memphis Area Engineering and Science Conference, MAESC.

[34] Kruse R., Mostaghim S., Borgelt C., Braune C., Steinbrecher M. (2022). Multi-layer perceptrons. Computational Intelligence: A Methodological Introduction.

[35] Chen T., He T., Benesty M., Khotilovich V., Tang Y., Cho H., Chen K., Mitchell R., Cano I., Zhou T. (2015). Xgboost: Extreme Gradient Boosting.

[36] Wiens M., Verone-Boyle A., Henscheid N., Podichetty J.T., Burton J. (2025). A tutorial and use case example of the eXtreme gradient boosting (XGBoost) artificial intelligence algorithm for drug development applications. Clin. Transl. Sci..

[37] Mohammadi S., Eliassen F., Zhang Y., Jacobsen H.A. (2022). Detecting False Data Injection Attacks in Peer to Peer Energy Trading Using Machine Learning. IEEE Trans. Dependable Secur. Comput..

[38] Mohammadi S., Eliassen F., Zhang Y., Jacobsen H.A. (2021). Attack dataset Austin Texas 2018. IEEE Dataport.

[39] Fu Y., Liu D., Chen J., He L. (2024). Secretary bird optimization algorithm: A new metaheuristic for solving global optimization problems. Artif. Intell. Rev..

[40] Xu W., Kobayashi K., Yamashita Y. (2025). Cyber Attack Detection in P2P Energy Trading Using Machine Learning. Proceedings of the 2025 IEEE 14th Global Conference on Consumer Electronics (GCCE).

[41] Shi Y., Eberhart R. (1998). A modified particle swarm optimizer. Proceedings of the 1998 IEEE International Conference on Evolutionary Computation.

